# A Computational Quantum-Based Perspective on the Molecular Origins of Life’s Building Blocks

**DOI:** 10.3390/e24081012

**Published:** 2022-07-22

**Authors:** Gabriele Amante, Judit E. Sponer, Jiri Sponer, Franz Saija, Giuseppe Cassone

**Affiliations:** 1 Department of Mathematical and Computer Science, Physical Sciences and Earth Sciences, Università degli Studi di Messina, V. le F. Stagno d’Alcontres 31, 98166 Messina, Italy; amantegabriele@gmail.com; 2 Institute of Biophysics of the Czech Academy of Sciences (IBP-CAS), Kràlovopolskà 135, 61265 Brno, Czech Republic; judit@ncbr.muni.cz (J.E.S.); sponer@ncbr.muni.cz (J.S.); 3 Institute for Physical-Chemical Processes, National Research Council of Italy (IPCF-CNR), V. le F. Stagno d’Alcontres 37, 98158 Messina, Italy

**Keywords:** prebiotic chemistry, astrobiology, origins of life, ab initio molecular dynamics, metadynamics, density functional theory

## Abstract

The search for the chemical origins of life represents a long-standing and continuously debated enigma. Despite its exceptional complexity, in the last decades the field has experienced a revival, also owing to the exponential growth of the computing power allowing for efficiently simulating the behavior of matter—including its quantum nature—under disparate conditions found, e.g., on the primordial Earth and on Earth-like planetary systems (i.e., exoplanets). In this minireview, we focus on some advanced computational methods capable of efficiently solving the Schrödinger equation at different levels of approximation (i.e., density functional theory)—such as ab initio molecular dynamics—and which are capable to realistically simulate the behavior of matter under the action of energy sources available in prebiotic contexts. In addition, recently developed metadynamics methods coupled with first-principles simulations are here reviewed and exploited to answer to old enigmas and to propose novel scenarios in the exponentially growing research field embedding the study of the chemical origins of life.

## 1. Introduction

1953 may be considered as an *annus mirabilis* for our comprehension of fundamental aspects related to life as we know it and its chemical origins. In fact, during that year, two pioneering and outstanding papers were published in *Nature* [1] and in *Science* [2] journals. Whereas the former, reported by Watson and Crick [1], unveiled the molecular unit containing all the necessary information for the functioning of all living beings (i.e., the deoxyribonucleic acid, DNA), the latter—gone down in history as the Miller–Urey experiment [2]—succeeded in the identification of some plausible chemical pathways leading to the first biogenic molecules from simple organic and inorganic precursors.

The Miller–Urey experiment [2] posed the basis for the onset of a novel scientific branch currently known as prebiotic chemistry, based on the theoretical hypothesis of the chemical evolution proposed in the thirties of the previous century separately by Alexander I. Oparin and John B. S. Haldane [3]. This relatively young research field encompasses multi- and interdisciplinary chemical approaches aimed at investigating the fundamental mechanisms underlying the transformation of simple molecular units into more complex species directly associated with the onset of life [4]. Since 1953, a rather conspicuous number of papers were published proposing various prebiotic environments and different energy sources potentially compatible with realistic primordial scenarios [5,6,7,8,9,10,11,12,13,14,15]. Additionally to specific molecular building blocks, indeed, peculiar catalytic energy supplies (i.e., intense electrical discharges typical of lightning phenomena, UV light, strong mechanical stresses generated under meteorite impacts, high-pressure/high-temperature regimes found in hydrothermal vents, etc.) are necessary to trigger potentially relevant prebiotic reactions [16,17,18,19,20,21]. These energy sources were essential to drive a set of highly diverse simple molecules bearing elements such as hydrogen, oxygen, carbon, nitrogen, and phosphorus (just to cite the most abundant ones) toward complex biological molecules such as DNA and ribonucleic acid, RNA.

Although DNA and RNA are stable molecules capable of storing genetic information, the presence of the hydroxyl group on the 2${}^{\prime }$ carbon of the ribose of RNA (and its absence on the 2${}^{\prime }$ carbon of the deoxyribose of DNA) renders this latter a more reactive species with respect to DNA [22,23]. This evidence, along with other important aspects, led to the proposal of the “RNA-first” or “RNA-world” hypothesis where—rather than DNA—RNA is considered the pivotal molecular precursor of the first living beings appeared on our planet [12]. In fact, the lower stability accompanied by its self-replicating capability makes RNA a better candidate than DNA in the prebiotic realm. However, there is the question of how the RNA and DNA building blocks such as sugars and nucleobases, along with amino acids ascribed to life, were primordially synthesized. This latter question represents the key enigma that prebiotic chemistry tries to answer [24,25].

As anticipated, the Miller–Urey experiment [2] reported on the first experimental evidence of the possibility to convert simple inorganic and organic molecules, widespread in Earth-like primordial atmospheres, into prebiotic building blocks of life-giving molecules upon exposure to electric discharges mimicking lightning phenomena. Since then, a plethora of other experiments were performed (see [26,27] and references therein) not only with the aim of understanding the generality of the Miller–Urey experiments but also by employing diverse starting compounds such as hydrogen cyanide (HCN). As shown by Orò [8,9], indeed, a concentrated aqueous solution of ammonium cyanide (the ammonium salt of HCN), is capable of undergoing multiple chemical transformations affording adenine—one of the nucleobases—by simple heating. Moreover, in addition to amino acids and nucleobases, the primordial formation of sugars—within the so-called formose reaction [28,29,30]—also holds a pivotal role in the seminal steps of early molecular evolution leading to RNA. At the same time, some hypotheses proposed that other simple molecules could have served as feedstock molecules in prebiotic synthetic processes. One of these suggests that formamide (H_2_NCHO) could have accumulated in sufficiently high amounts to serve as the building block and the reaction medium for the synthesis of the first biogenic molecules [14,31].

Notwithstanding all these experimental evidences established the foundation and the playground of prebiotic chemistry, owing to the exponential growth of the supercomputing power [32,33] nowadays all experimental procedures are routinely supplemented by numerical simulations. These computations are capable of shedding light on the molecular mechanisms governing the transformation of simple molecules into more complex ones under reliably simulated prebiotic scenarios. In particular, by exploiting the robust computational toolkit stemming from quantum mechanics and density functional theory (DFT), traditional static quantum chemical calculations and advanced ab initio molecular dynamics simulations have recently been capable of reintroducing potentially relevant prebiotic molecules—such as, e.g., formamide itself—in the whole framework concerning the chemical origins of life. In fact, although formamide as a prebiotic molecule was already considered by early experimental studies in the 1970s, due to the works of Schoffstall [34] and Yamada et al. [35] and reconsidered 20–30 years later by Saladino and Di Mauro [31], only during the last decade computations have been capable of corroborating the prebiotic role of this key intermediate species [36,37,38,39,40,41,42,43,44,45,46]. Understanding this new conceptual framework where it is feasible to monitor all the electronic, atomic, and molecular processes involved in prebiotic chemical reactions owing to the exploitation of quantum mechanical calculations, makes it possible not only to test new hypotheses but also to deepen and accelerate our comprehension of the general patterns behind the complexity of the chemical origins of life.

In the current minireview, we outline the key findings obtained in selected investigations employing supercomputing approaches that recently enabled the microscopic understanding of the onset of the building blocks of life, such as amino acids and sugars, not only under plausible prebiotic conditions but also in the whole Universe.

## 2. Methods

### 2.1. Theory

Lightnings, with their associated strong electrostatic potential gradient, are capable to trigger several transformations in matter at the molecular level, also of prebiotic interest. From a genuine theoretical and computational perspective, the inclusion of the catalytic effects triggered by the application of intense electric fields on matter was problematic since the origins. In fact, since the definition of polarization, a long story witnesses the efforts devoted for developing and implementing methods suitable for the description of electric fields in density functional theory (DFT) algorithms [47,48,49,50,51,52].

The toughest problem is posed by the periodicity of the simulation box. Because of the nonperiodic quantum position operator, periodicity in presence of an electric field E leads to a significant modification of the electron potential in all replica of the simulation box. Incidentally, the ground state is also ill-defined [50,53,54]. Several perturbative approaches for the implementation of electric fields were reported, but only owing to the Modern Theory of Polarization and Berry’s phases such a tricky problem was successfully solved [48,49,55], as also summarized by some of ours in [56]. Alternatively, also the effective screening medium (ESM) method, which precisely overcomes this issue—especially when simulating charged electrodes surfaces—may be invoked [57]. On the other hand, following the Modern Theory of Polarization, Nunes and Gonze [51] proved that perturbative analyses can be straightforwardly earned from a variational principle relying on the minimization of the following functional

(1) $$F=E_{KS}\left(\left\{\psi_{kn}\right\}\right)-E\cdot P\left(\left\{\psi_{kn}\right\}\right).$$

In this equation, $E_{KS}\left(\left\{\psi_{kn}\right\}\right)$ is the Kohn–Sham (KS) energy depending on the occupied Bloch’s functions while $P(\left\{\psi_{kn}\right\})$ represents the zero-field Berry’s phase of the polarization associated with the electrons.

Within the KS scheme, Berry’s phase polarization of the noninteracting KS system is not exact [58,59]. A strategy for circumventing this issue is that of taking into consideration a more general Hohenberg–Kohn theorem where the electron density n(r) and the polarization P(r) feature in a unique manner all ground-state properties [58,59]. The authors of [47] admirably demonstrated that the functional (1) is usable as energy functional for a variational approach also within the finite-field scenario. This way, the computation of the polarization **P** under the action of electric fields affords the solution to the problem of calculating all properties of an insulator in an homogeneous electric field. Indeed, the inclusion of Berry’s phase [55] polarization into Equation (1) solves the problem. More specifically, by considering an electric field along a given direction, the following energy functional can be written

(2) $$E^{E}\left[\left\{\psi_{i}\right\}\right]=E^{0}\left[\left\{\psi_{i}\right\}\right]-E\cdot P\left[\left\{\psi_{i}\right\}\right]\;,$$

where $E^{0}\left[\left\{\psi_{i}\right\}\right]$ is the energy functional in absence of the field and $P[\left\{\psi_{i}\right\}]$ is the polarization defined by [52]:

(3) $$P\left[\left\{\psi_{i}\right\}\right]=-\frac{L}{\pi }Im\left(\ln detS[\left\{\psi_{i}\right\}]\right)\;,$$

where *L* is the periodicity of the system (i.e., the edge of the simulation box) and $S[\left\{\psi_{i}\right\}]$ is

(4) $$S_{i,j}=\langle \psi_{i}|e^{2\pi ix/L}|\psi_{i}\rangle .$$

Such a formulation can be extended to also yield the forces. By implementing the following quantity to (2)

(5) $$E_{ion}^{E}=-E\cdot P_{ion}\;,\;\;\;\;P_{ion}=\sum_{i=1}^{N_{ion}}Z_{i}\cdot R_{i}\;,$$

where $P_{ion}$ represents the polarization associated with the nuclei, $R_{i}$ is the spatial coordinate along the axis of the field, whereas $Z_{i}$ is the charge stemming from the protons in the nuclei. Clearly, such a specific expression generates a component to the force present on the *i*th atom, corresponding to  $F_{i}=EZ_{i}$. Such a pioneering theoretical framework [47] has been extensively used in most of the computations summarized in the current minireview.

Another sophisticated computational method employed in the works summarized in the current minireview deals with the modeling of the propagation of single as well as multiple shock waves in matter systems. This approach is known as the multiscale shock-compression simulation technique (MSST) [60] and it is based on the Euler equations for compressible flow. Such equations permit the conservation of mass, momentum, and energy in all points belonging to the wave. One of the major strengths of this approach is the possibility of exploring the relevant configuration space within the timescales affordable by common molecular dynamics simulation techniques. In fact, the MSST allows for the dynamical simulation of condensed-phase systems under dynamical shock conditions for orders of magnitude longer time periods than possible using standalone nonequilibrium molecular dynamics approaches, offering a unique computational advantage in computationally demanding simulations such as first-principles molecular dynamics. Last but not least, this approach relieves typical finite-size issues related with the simulation box, while at the same time allowing for a realistic description of the chemistry under extreme conditions, a unique combination of computational benefits rendering this advanced technique perfectly suited for the simulation of meteorite impacts, dust grain collisions, and other catastrophic mechanical events plausible for the onset of prebiotically relevant chemical species [36,39].

### 2.2. Simulations

Computations and numerical simulations discussed in this minireview were executed either exploiting the Car–Parrinello [61] (CPMD) approach or by using Born–Oppenheimer molecular dynamics (BOMD) techniques, which fall under the umbrella of the ab initio molecular dynamics (AIMD) methods. The software suite Quantum ESPRESSO [62] was employed when running CPMD simulations. On the other hand, when the BOMD formalism was adopted by us, the software package CP2K [63,64] was used. In all cases where the effects triggered by lightning phenomena were investigated, several liquid samples of disparate chemical nature were posed under the action of static and homogeneous electric fields applied along a given direction. As usual, for the sake of comparison, several prolonged AIMD simulations were always performed at zero field.

In all cases in which a uniaxial shock compression was applied to investigate a potential impact-induced chemistry, the multiscale shock-compression technique (MSST) [60] was adopted. The shock wave was propagated along the *x*-axis of the employed reference system. After compression, decompression of the simulation boxes enabled the analysis and identification of the most stable chemical reaction products. We tested uniaxial shock-wave velocities ranging from 6 to 10 km·$s^{-1}$ producing several shock-compressed thermodynamic states by means of the MSST in trajectories having an approximate length of about 5–10 ps. The most compressed state was then decompressed toward its final decompressed pressure corresponding to the unperturbed (starting) simulation cell.

As for all the specific technical details on the numerical boxes simulated in the investigations here reported, we kindly invite the interested reader to refer to the original publications (see, e.g., [18,38,39,40]). The investigated samples include many different kinds of liquids, from systems replicating the original Miller–Urey samples [38] to aldehyde aqueous solutions [40], and from prototypical simple mixtures present in dust grains [39] to planetary atmospheres compositions [18]. When an electric field was applied, the intensity of the electric field was gradually increased with a step increment of 0.05 V/Å from zero up to ∼0.50 V/Å. Albeit nuclear quantum effects may play a role in assisting specific electric-field-driven chemical reactions [65], in all cases here summarized the dynamics of the nuclei was treated classically by invoking the Verlet algorithm. Canonical sampling has generally been executed in CPMD simulations through the coupling of the system under investigation to the Nosé–Hoover thermostat or, during BOMD computations, by exploiting CSVR thermostats [66].

Several DFT exchange-correlation functionals were employed depending on the specific system under investigation. Among them, the Perdew–Burke–Ernzerhof (PBE) [67] and the Becke–Lee–Yang–Parr (BLYP) [68,69] functionals represented the most frequently employed ones. In addition, with the aim of taking into account dispersion interactions, semiempirical corrections (i.e., PBE-D3 and BLYP-D3) [70,71] were used. While in most of the CPMD simulations, cutoff energies of 35 Rydberg (Ry) and 280 Ry allowed us to adopt timesteps of ∼0.10–0.12 fs, in BOMD simulations, plane–wave cutoffs of 400 Ry were generally imposed. Moreover, as usual for BOMD, timesteps larger than those used for CPMD were almost always set (i.e., 0.50 fs). Whereas in CPMD, the core electronic interaction was treated via ultrasoft pseudopotentials (USPP), in BOMD, Goedecker–Teter–Hutter pseudopotentials using the GPW method were generally employed. In AIMD, numerical calculations were executed by means of the CP2K software and the wavefunctions of the atomic species were expanded in triple-zeta valence plus polarization (TZVP) basis sets, whereas during CPMD simulations, only plane waves were employed.

Albeit AIMD simulations represent one of the most efficient computational tools for mimicking the chemical nature of matter, the timescale of current AIMD simulations are limited by the relatively high computational demand of quantum-based computations. As a consequence, determination of accurate free-energy surfaces (FES) is rather difficult by means of AIMD simulations only. On the other hand, there exists an efficient numerical approach—known as metadynamics (MetD) [72]—which is able to accelerate a given process, allowing the system to visit the most likely stability valleys. At the same time, MetD reproduces the free-energy landscape of the process. In order to find smart and general reaction coordinates, a path Collective Variables MetD [73], was developed and an updated version of it has been reported [74,75]. This powerful method has been exploited owing to the usage of PLUMED [76,77] to quantitatively determine the free energy associated with the first fundamental reaction step of the formose reaction [40] and to reproduce the conversion of glycine into ethanolamine at different pressures.

## 3. Results and Discussion

### 3.1. Amino Acids Synthesis: The Miller-Urey Experiments

Being the first experiment succeeding in the identification of some plausible chemical pathways leading to the first biogenic molecules from simple organic and inorganic precursors, the Miller–Urey experiment is considered the progenitor of the research branch currently known as prebiotic chemistry. The original Miller–Urey experimental setup was composed of a flask where water was boiled. The formed water vapor could evaporate, through a tube, into another flask containing methane (${\mathrm{CH}}_{4}$), ammonia (NH_3_), and molecular hydrogen (H_2_). This mixture was exposed to a strong electrostatic field generated by a Tesla coil simulating the intense lightning phenomena plausibly present in the relatively unstable primordial atmosphere. After such a step, the gas was condensed into the first flask and cyclically subjected to the same process. A recent reproduction of the Miller–Urey experiments, highlighting the (previously overlooked) role played by the borosilicate glasses composing the surfaces of the flasks, was conducted by Saladino, Di Mauro, and coworkers [78].

After one week since the beginning of the experiment in 1953, Miller observed a visible change of the color of the original mixture. In fact, the latter took a red coloring, suggesting that some chemical transformations occurred in the samples. By analyzing the produced mixture via paper chromatography, it was evident that α-alanine, β-alanine, and glycine—the simplest amino acid—were formed during the process. This seminal experiment revealed, for the first time, that simple, inorganic and organic, and relatively inert species could be directly transformed into prebiologically relevant molecules such as amino acids by means of the simple exposure of the original samples to an ubiquitous energy source, abundantly available in the primordial terrestrial atmosphere [6,20,79].

Intuitively, Miller and Urey proposed that in their experiment, the amino acid products form according to the Strecker synthesis [5]. This latter is a chemical process in which amino acids are synthesized through α-amino nitriles that form by the reaction of simple aldehydes with ammonia (NH_3_) and hydrogen cyanide (HCN). On the other hand, the experimental apparatus they exploited was not adequately equipped to prove the hypothesized elementary reaction steps. Indeed, presence of amino nitriles or HCN was not detected in the reaction products. After all, on-the-fly tracking of fast chemical reactions occurring on timescales on the order of hundreds of femtoseconds (fs) represents a hard task also for modern-day reaction kinetics studies. By contrast, as it will be laid out in the following, this kind of information is achievable by means of sophisticated and robust first-principles simulations.

Ab initio molecular dynamics (AIMD) approaches are indeed particularly useful when investigating specific physical and chemical transformations taking place at a submicroscopic level providing a complete picture of all the steps underlying given chemical reactions. In 2014, about 60 years after the original experiments, the in silico reproduction of the Miller–Urey experiments was reported for the first time [38] by exploiting the powerful tools provided by Car–Parrinello molecular dynamics techniques [61]. One of the main findings of this work was that of proving the key role played by some intermediate species of the likes of formic acid (HCOOH) and formamide (H_2_NCHO) in assisting the conversion of the simple and quite inert molecules employed by Miller and Urey (i.e., H_2_O, CH_4_, NH_3_, H_2_, etc.) into the simplest amino acid glycine [38]. In fact, it turned out that the formation of molecular species such as formaldehyde (H_2_CO) and hydrogen cyanide (HCN), previously and commonly thought to be fundamental for the Strecker synthesis of amino acids from the Miller–Urey samples [5], is an energetically unfavored process at the level of theory at which these mechanisms were explored (i.e., density functional theory (DFT) at the generalized gradient approximation (GGA) without dispersion interactions). Interestingly, from a series of unbiased Car–Parrinello molecular dynamics simulations, a completely different route heading toward the synthesis of prebiotically important molecules has been identified in this work. In particular, one molecular hydrogen, one carbon monoxide, and one ammonia molecule recombined during these simulations under the action of externally applied static and homogeneous electric field strengths on the order of 0.50 V/Å to directly produce a formamide molecular species. Incidentally, such a molecule represents a key intermediate found in independent prebiotic reactions taking place under multiple circumstances [17,39,80,81]. Moreover, additionally to formamide, in a few steps of Car–Parrinello dynamics under these extreme external conditions, the synthesis of 2 formic acid species obtained via the concerted recombination of an hydroxide anion (OH)${}^{-}$ with a carbon monoxide CO species neutralized by a proton H^+^ diffusing across the hydrogen-bond network of the liquid mixture by means of the typical Grotthuss mechanism was directly observed [38].

During this kind of numerical experiments, the external electric field is gradually increased. In the in silico reproduction of the Miller–Urey experiment, when the field intensity reached 0.50 V/Å, all the relevant chemistry also observed in the laboratory experiments took place. In fact, once formed, formamide then either fueled the synthesis of larger and more complex molecules, or broke down into water and hydrogen cyanide, as observed in the standard Strecker reaction [5,6,79], and as detailed in the following:

(6) $$\begin{array}{cc} NH_{3}+CO & \overset{E∼0.5\;V/\overset{˚}{A}}{\to }H_{2}NCHO\\ \; & \overset{E∼0.5\;V/\overset{˚}{A}}{\to }H_{2}O+HCN \end{array}$$

In parallel, a quite less plausible reaction was observed between water and carbon monoxide, which gives rise to formic acid:

(7) $$\begin{array}{cc} H_{2}O+CO & \overset{E∼0.5\;V/\overset{˚}{A}}{\to }HCOOH. \end{array}$$

After few steps of Car–Parrinello molecular dynamics, a carbon monoxide combined with a formamide molecule, a complex that spontaneously broken into a hydrogen cyanide and a formate anion. These latter combined with a formamide-proton cation to yield a α-hydroxyglycine:

(8) $$\begin{array}{cc} {\left(H_{2}NCHOH\right)}^{+}+{\left(COOH\right)}^{-} & \overset{E∼0.5\;V/\overset{˚}{A}}{\to }H_{2}N-CHOH-COOH. \end{array}$$

Once formed, α-hydroxyglycine evolved into glycine [38], proving the key catalytic role played by the external electric field and the power of DFT-based simulations in disclosing otherwise-difficult-to-achieve information.

**Figure 1:**
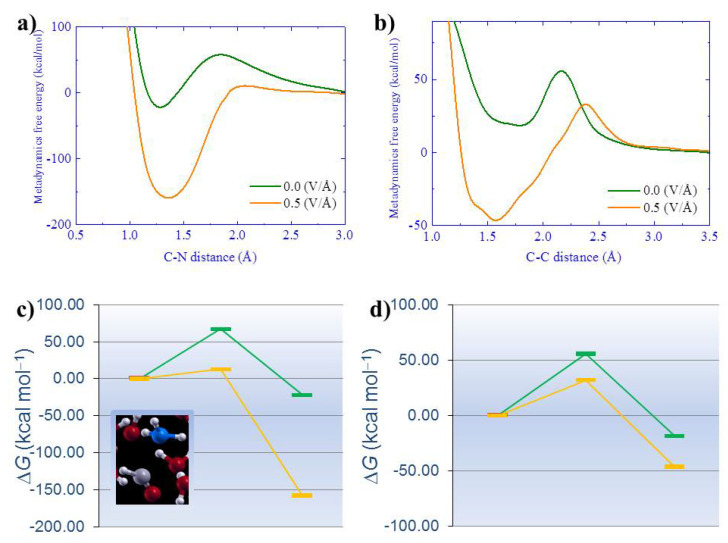
Metadynamics-based free-energy profile as a function of the carbon-to-nitrogen (C-N) (**a**) and carbon-to-carbon (C-C) (**b**) distance reaction coordinate. In the bottom panel, the energetics of the reaction between C-N (**c**) and C-C (**d**) with (orange line) and without (green line) the electric field are presented. Energy values are expressed in kcal·${\mathrm{mol}}^{-1}$.

Another pivotal result afforded by those simulations was the attempt to quantitatively measure the thermodynamic supply provided by the external field during the observed chemical reactions. In fact, most of the detected chemical transformations occurred on timescales on the order of 2–3 picoseconds (ps) and were accompanied by a potential energy drop, indicating favorable exothermic mechanisms [38]. In addition, some preliminary simple metadynamics calculations were executed in this seminal work to figure out the free-energy contribution carried by external static fields when some crucial carbon-to-nitrogen (Figure 1a,c) and carbon-to-carbon (Figure 1b,d) are formed in the system, as shown in Figure 1.

Although these additional simulations showed that the application of an electric field is capable of decreasing the height of the free-energy barrier, the fact that a significant finite barrier still persists at a strong field intensity of 0.50 V/Å witnesses that the choice of the simple interatomic distance between species forming either C-N or C-C bonds does not represent a good choice for the reproduction of the complex multidimensional free-energy landscape. This way, additional simulations performed by means of metadynamics techniques clarified some of these crucial aspects [82]. Further DFT-based and metadynamics simulations were executed in direct connection with more recent Miller–Urey-like experiments by Ferus et al. [20].

A marginal note is finally devoted to the role held by the presence of ionic species created by the action of the field. In fact, all the presented reactions were not caused by the presence of ions in solution but is the field itself to be the key ingredient in all the prebiotic processes described in this section. In fact, by running a series of simulations of the same initial set of the Miller–Urey molecules in complete absence of the external electric field but replacing all water and ammonia molecules with water and ammonia ion, no significant reaction with exception of trivial neutralization/recombination processes were observed [38], proving the fundamental role of the electrostatic field, as also more recently proven by investigating the electrochemical behavior of glycine and alanine on a biased platinum surface by quantum electrochemistry [46]. Obviously, it is not possible to rule out that in real systems, radical species could contribute to the observed reactivity; such factors are not satisfactorily considered in DFT calculations. On the other hand, all these results confirm that the electric field is not just useful to dissociate molecules into ions but that it also represents an order-maker agent favoring the assembling of larger chemical units from smaller ones, and hence affording chemical complexity.

### 3.2. Sugars Synthesis: The Formose Reaction

The formose (*portmanteau* of **form**aldehyde and ald**ose**) reaction is a series of relatively complex autocatalytic reactions yielding sugars from simple aldehydes. It was discovered in 1861 by Butlerov, almost a century before the prebiotically relevant results earned by Miller and Urey. Thus, at the time of the discovery of the formose reaction, neither connections nor implications were made to the prebiotic chemistry framework, also because the existence and the chemical structure of DNA and RNA were not known yet. Since both DNA and RNA have a “sugarous” backbone, it is likely that the formose reaction could have played a role toward the synthesis of sugars within the chemical origins of life paradigm.

Since its discovery, many scientists proved many of the (almost) endless possible pathways that this complex chemical reaction network can undergo. The conversion of simple aldehydes into simple sugars was first observed in a system containing formaldehyde in an alkaline solution [28]. Under these conditions, the formose reaction occurs spontaneously and yields multifaceted mixtures of sugars. These latter, however, further reacts to form an insoluble polymeric material, even though ribose could be selected by borate minerals [83]. Another circumstance in which this process experimentally takes place is in presence of catalytic clays capable—similarly to the alkali-catalyzed formose reaction—to yield sugars in a nonselective manner. For instance, it has recently been shown that UV irradiation of astrophysically relevant ice mixtures containing simple species such as ammonia (NH_3_), methanol (CH_3_OH), and water (H_2_O) leads to the formation of aldehydes and of simple—prebiotically relevant—sugars such as glycolaldehyde and glyceraldehyde [30]. These latter are, indeed, among the possible chemical intermediates in the ribonucleotide synthesis.

As mentioned in the Introduction, RNA is assumed to be the first biological molecule appeared on our planet within the so-called “RNA-world” hypothesis. This is mainly due to the presence of the sugar ribose in its backbone, which confers to RNA a sufficiently high reactivity enabling this molecule to perform a catalytic function. This latter feature may have held a key role during the first molecular evolutive steps toward more complex entities. Thus, understanding how ribose and its multiple precursors could have been produced under primordial Earth-like conditions is of primary interest in prebiotic chemistry and astrobiology. It is well established from several laboratory experiments that ribose can be formed only in modest quantities during different types of formose reactions and that it quickly caramelizes to an insoluble tar [29]. More recent investigations have proved the possibility to conduct a whole formose reaction up to ribose and ribose-related compounds under UV radiation [84]. Due to the brief lifetime of ribose observed in some experiments, other “XNA theories”, involving simpler RNA precursors formed by either a threose or a peptide backbone capable of storing and transcribing genetic information but holding a simpler and more stable moiety, have been proposed [85,86,87,88].

Notwithstanding the presented difficulties in executing a formose reaction, the most famous weakness pertaining to the traditional view of the formose reaction is represented by the formation of the first carbon-to-carbon (C–C) bonds from formaldehyde (H_2_CO)—the simplest aldehyde—which represents the rate-limiting step of the formose reaction. In fact, in order to observe the reaction leading from formaldehyde to glycolaldehyde

(9) $$\begin{array}{cc} 2H_{2}CO & \overset{}{\to }HOCH_{2}CHO\;, \end{array}$$

the so-called *umpolung* (i.e., the polarity inversion) of formaldehyde should occur, which is manifestly a thermodynamically and chemically disfavored process, independently from the boundary operational conditions. Although such an evidence is quite manifest, only in the last years it has been possible to calculate via ab initio molecular dynamics and advanced metadynamics methods exploiting path Collective Variables as reaction coordinates [74,89] the free energy associated with the *umpolung* event [40]. It emerged that to synthesize a glycolaldehyde molecule (HOCH_2_CHO), the simplest sugar (a diose) and the second simplest aldehyde after formaldehyde, in a formaldehyde aqueous solution, an initial release of protons is required from one of the formaldehyde molecules [40]. To cross the free-energy barrier separating those basins, +30 kcal·mol^−1^ first have to be invested. Furthermore, the synthesis of glycolaldehyde takes place only by climbing an uphill and steep free-energy surface connecting two metastability basins having a +40 kcal·mol^−1^ difference. Though this latter free-energy barrier is about 10 kcal·mol^−1^ lower than that found for its gas-phase counterpart [90], advanced quantum-based molecular dynamics simulations have quantitatively proved why the first step of the formose reaction (i.e., the conversion of formaldehyde into glycolaldehyde) is definitely unlikely [40].

**Figure 2:**
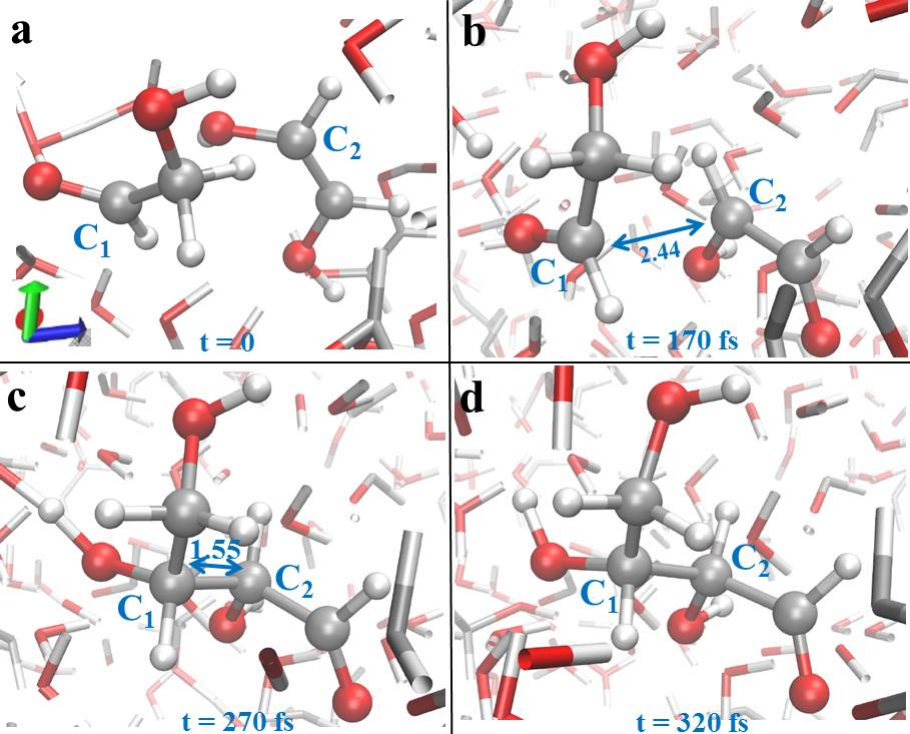
Formation of (D)-erythrose as reported and detailed in [40]. Carbon-to-carbon (C-C) distances are highlighted in Å. When an aqueous solution of glycolaldehyde was exposed to a static and homogeneous electric field of 0.45 V/Å, a newly synthesized 1,2-ethenediol reacts with a glycolaldehyde species (**a**) giving birth to a glycolaldehyde enolate (**b**). This way, the creation of a novel C-C bond takes place (**c**) and a (D)-erythrose molecule is synthesized rapidly (**d**).

As discussed in the previous sections, the application of intense electric fields on hydrogen-bonded systems significantly enhances the proton transfer activity [38,65,91,92,93,94] and, more interestingly, is able to open otherwise difficult-to-achieve reaction pathways [89,92,95,96,97]. In addition, it is well-known that protons are fundamental in lowering the free-energy barrier of the hypothesized synthesis of glycolaldehyde in the gas phase [90]. Based on this knowledge and on the quantitative evidence that starting a formose reaction from formaldehyde is hampered by a disfavored free-energy landscape, Cassone et al. performed a de novo Miller–Urey-like experiment from glycolaldehyde aqueous solutions. Moreover, glycolaldehyde is ubiquitous in the universe and may have been delivered on the primitive Earth from extraterrestrial sources [90,98,99]. This way, simulation boxes containing glycolaldehyde aqueous mixtures were exposed to increasingly higher field strengths from zero up to ∼0.5 V/Å in a series of Car–Parrinello and Born–Oppenheimer molecular dynamics simulations [40]. The first chemical field-induced modifications in the system was recorded for a field strength of ∼0.3 VÅ, which inter alia corresponds to the water dissociation threshold as determined by DFT-based approaches [65,91]. Thus, at this field intensity, hydroxide (OH)^−^ and hydronium (H_3_O)^+^ ions are concomitantly present in the solution along with glycolaldehyde and water molecules. The presence of these two species creates local electric fields even stronger than those applied externally. In fact, it is well-established that fields larger than ∼1 V/Å are typically measured in proximity of water counterions [65,100,101] and that these latter enhance the overall chemical reactivity. In fact, starting from field intensities of ∼0.4 V/Å, a newly synthesized 1,2-ethenediol molecule is detected in the simulation box (Figure 2a) and an enol tautomer of glycolaldehyde is produced in the system owing to a proton transfer with the surrounding molecular environment, as shown in Figure 2b. Whereas the presence of such a new compound in the simulated numerical sample favors a proton transfer from the nearby glycolaldehyde molecule to the enolate, the simultaneous presence of the external electrostatic potential gradient triggers the shortening of the distance between the two species leading to the stabilization of the newly formed C-C bond (Figure 2c). This way, a fast proton recombination suddenly leads to the production of (D)-erythrose (Figure 2d), a tetrose sugar which is the direct precursor of the pentose ribose.

**Figure 3:**
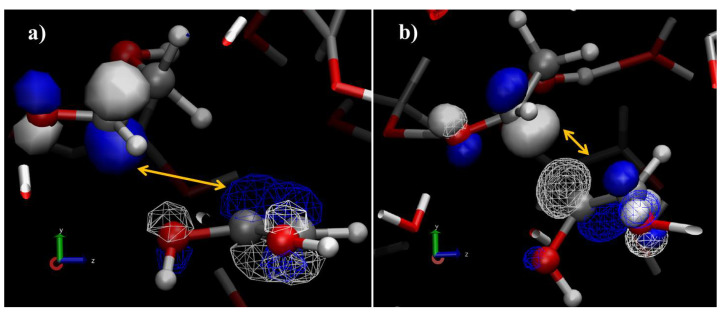
HOMO of the 1,2-ethenediol (**a**) transforming into a glycolaldehyde enolate molecule. (**b**) LUMO of the glycolaldehyde molecule, which lead to the (D)-erythrose formation through an aldol condensation chemical process. Wireframes mark the first HOMO of 1,2-ethendiol (**a**) subsequently transforming into a glycolaldehyde enolate (**b**). Solid surfaces identify the first LUMO of glycolaldehyde. White orbitals indicate positive electronic phases while blue orbitals identify negative phases for the electronic wavefunctions.

A key feature of these kind of advanced computational techniques is that they offer the possibility to track and directly visualize not only the behavior of the nuclei but also that of the electron densities to better interpret the field-induced formation of a new carbon-to-carbon bond in a system originally composed of glycolaldehyde and water, and also analyses of the kind of the frontier molecular orbital (FMO) may be exploited. By evoking the FMO theory [102], informative molecular orbitals such as the highest occupied molecular orbitals (HOMO) and the lowest unoccupied molecular orbitals (LUMO) of the two interacting molecules, which for a field strength of ∼0.5 V/Å yielded (D)-erythrose in a glycolaldehyde-water mixture, were determined [40]. The HOMO of the enol tautomer—subsequently evolving into a glycolaldehyde enolate species—and the LUMO of the neighbor glycolaldehyde molecule were evaluated, as shown in Figure 3. The most reactive HOMO of the 1,2-ethenediol species is delocalized all along the molecule, whereas the first LUMO of glycolaldehyde is located between the oxygen of the carbonyl group and the α-carbon (Figure 3a). Although this latter atom is close to the oxygen atoms of the 1,2-ethenediol, the confined spatial extent of the LUMO along with a nontailored phasing characterizing the interaction between the closest HOMO and LUMO prevents the formation of a carbon-to-oxygen covalent bond between these molecules (Figure 3a). Rapidly, indeed, (as highlighted in Figure 2), the system finds a configuration exhibiting the most expanded HOMO of the 1,2-ethenediol and the biggest LUMO of the glycolaldehyde as first-neighbor molecular orbitals which are in-phase. All these circumstances lead to the formation of the C-C bond producing the (D)-erythrose molecule [40].

In summary, advanced computational methods revealed that Miller–Urey-like experiments are capable not only to create amino acids but also to form sugars from simple and ubiquitous constituents. Again, the application of intense static electric fields is able to trigger the formation of new C-C bonds, a covalent bond which is at the basis of most biological species.

### 3.3. Meteorite Impacts: A Source of Chemical Complexity

Although lightning phenomena may have represented a key ingredient for the early molecular evolution of life in, e.g., planetary atmospheres, many other energy sources were available slightly before the onset of the first living organisms on our planet. In fact, in a period between approximately 4.1 to 3.8 billion years (Ga) ago—at a time corresponding to the Neohadean and Eoarchean eras on Earth—a huge number of asteroids collided with the early terrestrial planets in the inner Solar System, including Mercury, Venus, Earth, and Mars [103]. For instance, estimates of the Earth’s impact flux prior to ∼4 Ga suggest an annual energy deposition larger than 10^20^ J [104]. This enormous reservoir of collisional energy might have driven the complexification of small prebiotic feedstock molecules into biogenic compounds. Moreover, the fact that a plethora of violent shock impacts takes place in the whole Universe not only by means of collisions between asteroids and planetary surfaces but also between dust grains in dense molecular clouds indicates that the chemical complexity observed in some regions of the deep space might be the result of these peculiar—as well as ubiquitous—mechanical processes. The fact that glycine and other prebiotically relevant species were detected in non-negligible amounts in some comets, such as Halley, Hyakutake, Tempel-1, Giacobini–Zinner, Hartley 2 and Hale–Bopp, 81P/Wild 2 [105,106,107,108,109,110,111], indirectly suggested the possibility to observe and reproduce a relatively complex chemistry when high-energy impacts take place. In fact, many interesting experimental and computational results emerge from disparate investigations of the catalytic effects produced by violent mechanical stresses acting on simple inorganic systems [7,16,18,36,39,42,104,112,113,114,115,116,117,118,119,120,120]. As an example, Martins et al. [16] created several icy bullets composed of simple compounds ubiquitous in cometary ices such as ammonium hydroxide (NH_4_OH), carbon dioxide (CO_2_), and methanol (CH_3_OH), which were impacted on a series of rocky surfaces—mimicking planetary surfaces—by using a light gas gun available at an important facility located at the University of Kent [121]. This gun is able to shoot the samples at incredible speeds on the order of $v_{i}$∼7 km·$s^{-1}$, which corresponds to the mutual collisional speed of typical meteorite impacts. The shocked ice samples were then analyzed showing the presence of a series of amino acids, including glycine, (D)-alanine, (L)-alanine, and (L)-isovaline [16]. Similar experiments have more recently been performed by Singh et al. [120] at the same facility [121] by using more complex bullets composed of icy amino acids which, after being shot at a speed of ∼5 km·$s^{-1}$, have been transformed into complex biological structures such as peptides and membranelike structures.

A completely different kind of experiment on this topic is that typically performed by Ferus and coworkers (see, e.g., [42]). In fact, they exploit a high-power kJ-class laser system (PALS—Prague Asterix Laser System) with a pulse duration of 350 ps, a typical wavelength of 1315.2 nm, and possessing an energy of 150 J per pulse. Albeit under some specific operational configurations, PALS may be used to mimic lightning phenomena [122]; such a high-energy pulse creates in the shocked samples the thermodynamic conditions typical of those occurring in meteorite/cometary impacts, where a plume (i.e., a plasma) is locally produced due to the enormous collisional energy released. In one of these experiments, Ferus’ group focused such a powerful laser beam on a formaldehyde aqueous solution to mimic the formation of such a high-temperature plasma in presence of simple formaldehyde (H_2_CO) and water (H_2_O) molecules [42]. After a series of laser pulses, a Fourier transform infrared (FTIR) spectrometry clearly showed, on the one hand, the decomposition of formaldehyde into simpler species (i.e., carbon monoxide, carbon dioxide, methanol, etc.) and, on the other, the formation of interesting species such as 2-amino-2-hydroxy-acetonitrile and 2-amino-2-hydroxy-malononitrile [42]. As also proved by the same group in similar experiments, these latter molecules are key intermediate species in the formation of all RNA nucleobases (adenine, cytosine, guanine, thymine, and uracil) [19,123]. Of course, it is quite expensive and operationally complicated to setup experiments generating high-energy events and, with the aim of testing promising chemical pathways deserving to be further investigated in laboratory experiments, advanced computational techniques such as ab initio molecular dynamics coupled with the multiscale shock-compression technique (MSST) [60], are currently employed. As it will be pointed out in the following, these approaches are capable to directly reproduce and monitor the chemistry behind the transformation of inorganic into organic compounds upon violent shock-impact exposure of the original samples.

In a pioneering work exploiting crude (but computationally efficient) tight-binding density functional theory (TB-DFT) approximations, Goldman et al. [36] simulated the effects produced by shock impacts on a prototypical cometary ice mainly composed of ammonia, water, and methanol. These simulations not only showed that shock waves are able to drive the synthesis of transient C–N-bonded oligomers at extreme pressures and temperatures but also that upon quenching to lower pressures, these oligomers break apart to form a metastable glycine-containing complex [36]. Inspired by these findings and owing to a substantial improvement of the computational capabilities accomplished in that decade, Cassone et al. [39] simulated—by means of rigorous DFT-based molecular dynamics—the physical and chemical effects created during collisions between dust grains, where molecular hydrogen (H_2_) is certainly abundant and in presence of the simplest compound bearing all the primary biogenic elements: isocyanic acid (HNCO). In particular, such a mixture was subjected in a series of diverse simulations to unidirectional shock-wave compressions propagating at different speeds (i.e., 6, 7, 8, 9, and 10 km·$s^{-1}$). Although at 5 and 6 km·$s^{-1}$, no chemical changes were recorded, a significant strengthening of the intermolecular interactions was reported. By increasing the velocity up to 7 km·$s^{-1}$, the formation of new chemical species in the simulated box was observed. In particular, the synthesis of formamide and carbamoyl isocyanate was detected [39]. Even though only a small percentage of the reactant molecules was transformed, such an initial result not only indicated that some chemical complexity can be generated via meteorite impacts even in an extremely simple sample composed just of H_2_ and HNCO but also that formamide may act again as the springboard for further molecular complexification. As shown in Table 1, formamide and carbamoyl isocyanate are the only species formed also in the samples impacted by uniaxial shockwaves propagating at a speed of 8 km·$s^{-1}$ which, inter alia, produces an instantaneous Hugoniot temperature T_H_ equal to 1550 K and a peak pressure of 58 GPa in the most compressed state. However, a further increase of the velocity of collision triggers a pervasive chemical response in the simulated samples.

**Figure 4:**
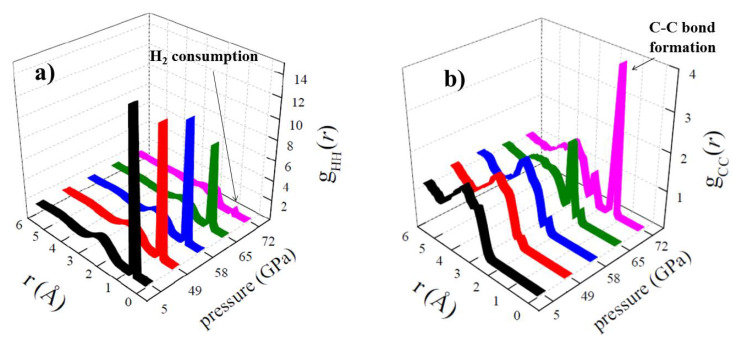
(**a**) Hydrogen-hydrogen and (**b**) carbon-carbon RDFs for different shock pressures, determined after decompression of the simulation boxes in ab initio molecular dynamics simulations coupled with the multiscale shock-compression technique.

As shown in Figure 4a, where the hydrogen-hydrogen (H-H) radial distribution functions (RDFs) are plotted, at peak pressures of 65 and 72 GPa—corresponding to shock speeds of 9 and 10 km·$s^{-1}$, respectively—most of the molecular hydrogen originally present in the system reacted to form some other species. Incidentally, the drastic reduction of the H-H RDF first peak at the most extreme conditions (10 km·$s^{-1}$, 72 GPa) coincides with the onset of a novel first peak in the carbon-carbon (C-C) RDF, as shown in Figure 4b. The fact that this new first maximum is located at ∼1.4 Å is the fingerprint of the birth of new C–C bonded species in the sample. As also listed in Table 1, the most important species formed are water, hydrogen cyanide, formic acid, glycine and other organic molecules—such as ethanimine (HNCHCH_3_) and vinylamine (H_2_NCHCH_2_)—which are the precursors to 7 amino acids: asparagine, aspartic acid, cysteine, leucine, phenylalanine, serine, and tyrosine. This way, it turned out that the transformation from chemical simplicity to chemical complexity can occur rapidly within the transient events following catastrophic impacts. Moreover, the fact that samples containing the most widespread molecule in the universe (H_2_) and the simplest compound bearing all of the primary biogenic elements (HNCO) can be transformed into glycine and amino acids precursors may suggest that the onset of complex structures ascribed to the birth of life as we know it may be a general process, ubiquitous in the whole universe.

Finally, we conclude the present minireview by presenting some original results showing that investigations on highly compressed molecular systems can be conducted via the coupling of ab initio molecular dynamics simulations with enhanced sampling techniques such as advanced metadynamics methods [72]. In particular, we have chosen an exemplary chemical reaction where glycine (H_2_NCH_2_COOH)—the simplest amino acid also found in the Murchison meteorite—is transformed into ethanolamine (H_2_NCH_2_CH_2_OH), a precursor of phospholipids recently detected in a molecular cloud in the interstellar medium [124]. Furthermore, amino alcohols are believed to stabilize short oligonucleotide sequences present in the prebiotic pool [125].

**Figure 5:**
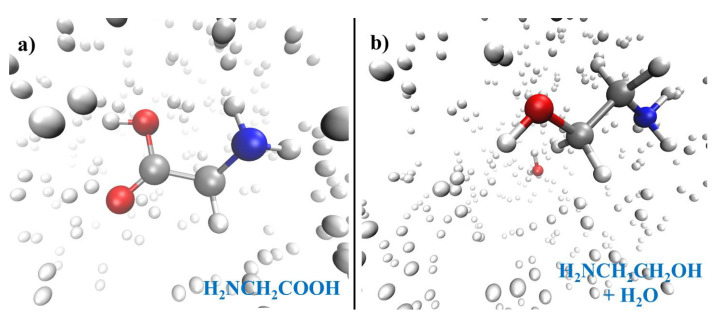
Snapshots of the simulation boxes containing glycine (**a**) and ethanolamine along with water (**b**) in compressed environments composed of molecular hydrogen.

**Figure 6:**
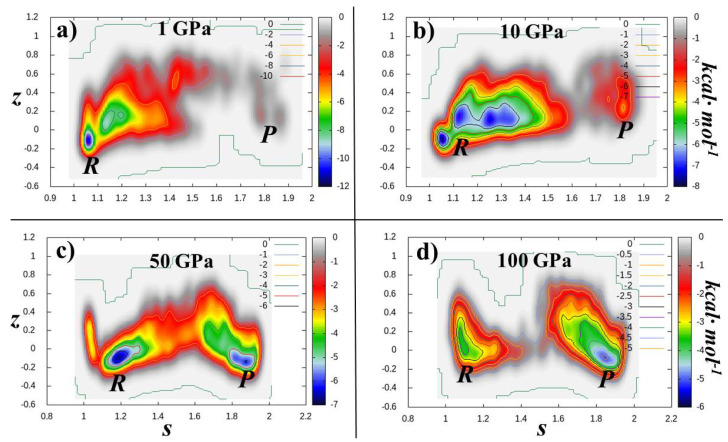
Free-energy landscapes determined from ab initio molecular dynamics and metadynamics methods of the conversion of glycine (reactants, R) into ethanolamine and water (products, P) in the space spanned by the path Collective Variables *s* and *z* (see text and Methods section) at pressures of 1 (**a**), 10 (**b**), 50 (**c**), and 100 GPa (**d**).

In particular, we have simulated the transformation of glycine into ethanolamine in a hydrogen-containing environment under different thermodynamic states corresponding to 4 different pressures (i.e., 1, 10, 50, and 100 GPa). The simulation box ascribed to the reactants can be visualized as depicted in Figure 5a and that of the reaction products is reported in Figure 5b. This way, whereas the dynamics of the atomic species has been simulated by means of DFT-based molecular dynamics, the conversion of the reactants into the products has been accelerated by means of a relatively recent metadynamics scheme [74,75,89]. Thus, by adding a history-dependent (Gaussian) potential to the relevant molecules participating to the reaction under investigation, the free-energy landscape of this chemical transformation has been reconstructed by employing two path Collective Variables, namely *s* and *z*. Whereas *s* follows the progress of the reaction from the reactants (to which it is typically associated a value of *s*∼1) to the products (to which it is typically associated a value of *s*∼2), the variable *z* is a measure of the orthogonal distance of the observed chemical pathway with respect to the idealized one. Although these simulations were carried out only with the aim of observing the chemical transformation without necessarily reach the convergence of the free-energy surface, they allow for a rough estimate of the free-energy supply necessary to the initial system to escape from its local free-energy minimum and achieve the product state, as shown in Figure 6.

It turns out that whereas under a modest (for an $H_{2}$ environment) pressure of 1 GPa, about 12 kcal·mol^−1^ have to be invested to transform glycine into ethanolamine (Figure 6a), only 3 kcal·mol^−1^ (Figure 6d) are required for running the same chemical reaction when an external pressure of 100 GPa—similar to those present during meteorite impact events—is imposed. Albeit such a difference may appear modest if compared to a two-orders of magnitude increase of the compression, it is worth noticing that according to the Eyring equation [126,127], a variation of the free-energy barrier height from 12 to 3 kcal·mol^−1^ at the same temperature is equivalent to an enhancement of 10${}^{9}$ of the kinetic rate constant of the investigated reaction, giving a better glance on the pressure-induced catalytic effects occurring, e.g., in violent impact events and under hydrothermal vent conditions.

## 4. Conclusions

In this minireview, we have reported a series of findings in the research fields of prebiotic chemistry and astrobiology. Additionally, to a general overview of some milestone historical and recent experiments, we have focused on advanced computational methods capable of efficiently solving the Schrödinger equation at different levels of approximation (i.e., density functional theory) and which are capable to realistically simulate the dynamical behavior of complex systems in condensed phase under the action of disparate energy sources available in prebiotic contexts.

In particular, a series of state-of-the-art numerical investigations elucidating the interplay between the quantum behavior of matter emerging at atomic and molecular scales with externally applied energy sources, such as intense electric fields mimicking lightning phenomena of primordial Earth-like atmospheres, violent shock impacts simulating the collision of meteorites with primitive planetary surfaces, and high-pressure/high-temperature regimes typical, e.g., of hydrothermal vents have been reported. Among them, ab initio molecular dynamics and enhanced sampling techniques (i.e., metadynamics) have been presented and exploited in the last decades to answer to old enigmas and to propose novel scenarios in the exponentially growing research field embedding the study of the chemical origins of life.

## Figures and Tables

**Table 1:** Inventory of species formed in the impact simulations of a simple H_2_+HNCO mixture under various conditions. *P*: maximum pressure reached in the ab initio simulation; $T_{H}$: maximum Hugoniot temperature reached; *v*: velocity of the uniaxial shockwave.

*P* (GPa)|$T_{H}$ (K)|*v* (km·$s^{-1}$)		
**37|857|6**	**58|1550|8**	**72|2122|10**
H_2_	H_2_	H_2_
HNCO	HNCO	CO_2_
	H_2_NCHO	H_2_O
	H_2_NCONCO	NH_3_, ${\mathrm{NH}}_{4}^{+}$
		HCN, CN^−^
		HCOOH
		H_2_NCHO
		CH_2_CHOH
		H_2_NCOOH
		CH_2_NH
		CH_3_NH_2_
		NH_2_CH_2_COOH
		CH_3_CHNH
		CH_2_CHNH_2_
		HNC(NH_2_)${}_{2}$ complex
		C-N aliphatic chains

## Data Availability

Data will be shared upon reasonable request to the authors.
